# Loss‐of‐function mutations in *ISCA2* disrupt 4Fe–4S cluster machinery and cause a fatal leukodystrophy with hyperglycinemia and mtDNA depletion

**DOI:** 10.1002/humu.23396

**Published:** 2018-01-22

**Authors:** Joseph T. Alaimo, Arnaud Besse, Charlotte L. Alston, Ki Pang, Vivek Appadurai, Monisha Samanta, Patroula Smpokou, Robert McFarland, Robert W. Taylor, Penelope E. Bonnen

**Affiliations:** ^1^ Department of Molecular and Human Genetics Baylor College of Medicine Houston Texas; ^2^ Wellcome Centre for Mitochondrial Research Institute of Neuroscience The Medical School Newcastle University Newcastle upon Tyne Tyne and Wear UK; ^3^ Royal Victoria Infirmary Great North Children's Hospital Newcastle upon Tyne Newcastle Upon Tyne UK; ^4^ Division of Genetics & Metabolism Children's National Health System Washington District of Columbia; ^5^ Department of Pediatrics The George Washington University School of Medicine and Health Sciences Washington District of Columbia

**Keywords:** encephalopathy, Fe‐S clusters, hyperglycinemia, ISCA2, leukodystrophy, mitochondrial dysfunction

## Abstract

Iron–sulfur (Fe–S) clusters are essential cofactors for proteins that participate in fundamental cellular processes including metabolism, DNA replication and repair, transcriptional regulation, and the mitochondrial electron transport chain (ETC). ISCA2 plays a role in the biogenesis of Fe–S clusters and a recent report described subjects displaying infantile‐onset leukodystrophy due to bi‐allelic mutation of *ISCA2*. We present two additional unrelated cases, and provide a more complete clinical description that includes hyperglycinemia, leukodystrophy of the brainstem with longitudinally extensive spinal cord involvement, and mtDNA deficiency. Additionally, we characterize the role of ISCA2 in mitochondrial bioenergetics and Fe–S cluster assembly using subject cells and *ISCA2* cellular knockdown models. Loss of ISCA2 diminished mitochondrial membrane potential, the mitochondrial network, basal and maximal respiration, ATP production, and activity of ETC complexes II and IV. We specifically tested the impact of loss of ISCA2 on 2Fe–2S proteins versus 4Fe–4S proteins and observed deficits in the functioning of 4Fe–4S but not 2Fe–2S proteins. Together these data indicate loss of ISCA2 impaired function of 4Fe–4S proteins resulting in a fatal encephalopathy accompanied by a relatively unusual combination of features including mtDNA depletion alongside complex II deficiency and hyperglycinemia that may facilitate diagnosis of ISCA2 deficiency patients.

## 1 INTRODUCTION

Iron–sulfur (Fe–S) clusters are sulfide‐linked iron molecules that serve as an essential cofactor for iron–sulfur proteins, which function throughout the cell to perform several fundamental cellular processes including transcriptional regulation, DNA and RNA synthesis and repair, electron transport, iron and heme metabolism, and oxygen sensing (Rouault, 2015; Stehling, Wilbrecht, & Lill, 2014). The generation of Fe–S clusters is an evolutionarily conserved multistep process involving over 15 proteins. However, the complete elucidation of the mechanism and machinery responsible for their biogenesis within each organism remains unresolved.

In humans, Fe–S cluster biogenesis is largely restricted to mitochondria for most Fe–S proteins including those that function in the mitochondria, cytosol, and nucleus (Lill et al., 2015; Rouault, 2015). There is evidence to suggest Fe–S biosynthesis may also occur independently in the cytosol and nuclear compartments, commonly referred to as the cytosolic Fe–S protein assembly (CIA) machinery that includes complexes for electron transfer, scaffolding, targeting and adapter function (Biederbick et al., 2006; Lill et al., 2015; Rouault, 2012, 2015; Tong & Rouault, 2006). The mitochondrial multiprotein Fe–S cluster (ISC) assembly machinery is responsible for de novo synthesis of clusters and assembles 2Fe–2S clusters onto scaffolding protein ISCU (Maio & Rouault, 2015; Stehling et al., 2014). Clusters may then be exported into the cytosol for further processing and integration into recipient iron sulfur proteins that ultimately function in the cytosol or nucleus or are retained within the mitochondria. 4Fe–4S clusters are formed from mature 2Fe–2S clusters within the mitochondria and molecular evidence has implicated a role for *ISCA1* (MIM# 611006), *ISCA2* (MIM# 615317), and *IBA57* (MIM# 615316) in this process (Muhlenhoff, Richter, Pines, Pierik, & Lill, 2011; Sheftel et al., 2012; Stehling et al., 2014). Characterization of these genes in *Saccharomyces cerevisiae* showed yeast orthologs *ISA1*, *ISA2*, and *IBA57* were essential for proper maturation of 4Fe–4S proteins (Gelling, Dawes, Richhardt, Lill, & Muhlenhoff, 2008; Melber et al., 2016; Muhlenhoff et al., 2011). Knockdown of either *ISCA1* or *ISCA2* in HeLa cells showed diminished activity of mitochondrial aconitase, complex I, and succinate dehydrogenase; the proper functioning of which rely on 4Fe–4S clusters (Sheftel et al., 2012). In contrast, HeLa cells depleted of *ISCA1* or *ISCA2* showed these genes were not required for ferrochelatase activity, which is a 2Fe–2S cluster protein. Structural characterization of *ISCA1* and *ISCA2* in vitro showed that an ISCA2 homodimer binds either 2Fe–2S or 4Fe–4S cluster and the ISCA1–ISCA2 heterodimer can bind two 2Fe–2S or 4Fe–4S clusters (Brancaccio et al., 2014).

A recent study described five independent consanguineous Arabic families with six affected children homozygous for a pathogenic missense variant in exon 3 of *ISCA2* (NM_194279.2 c.229G>A; p.Gly77Ser) (Al‐Hassnan et al., 2015). Phenotypic hallmarks included infantile‐onset developmental regression to a vegetative state, spastic tone, and optic atrophy. Characteristic cranial MRI findings did not include structural abnormalities, but rather extensive signal abnormalities in cerebral white matter and other regions including the cerebellum, middle cerebellar peduncles, and corpus callosum. Patient fibroblasts showed reduced copy number of mitochondrial DNA (mtDNA) and complex I enzyme activity at 20% of control, whereas other components of the electron transport chain (ETC) were not tested (Al‐Hassnan et al., 2015). Two unrelated patients with different novel homozygous missense variants in *ISCA2* (NM_194279.2 c.154C>T; p.Leu52Phe and c.313A>G; p.Arg105Gly) have been described, but with limited clinical information (Lebigot et al., 2017). Both patients’ fibroblasts showed a reduction of complex I activity and elevated complex III activity. Further analyses of these two patients showed conflicting results.

Here, we report the clinical description and functional characterization of two new, unrelated affected subjects who, like previously reported patients, are homozygous for the pathogenic *ISCA2* missense variant c.229G>A; p.Gly77Ser. We identified a profile of metabolic dysfunction that includes elevated CSF glycine and glutamate alongside low 5‐methyltetrahydrofolate. We provide a full functional characterization of mitochondrial bioenergetics, oxidative phosphorylation, and maintenance of the mitochondrial genome in tissue from patients harboring this founder mutation. Additionally, using both patient tissues and a human cell‐based *ISCA2* depletion model, we provide further support for the role of ISCA2 in the production of 4Fe–4S cluster proteins.

## 2 MATERIALS AND METHODS

### 2.1 Subjects

Informed consent for research studies was obtained in accordance with protocols approved by local Institutional Review Boards. Genomic DNA was extracted from peripheral‐blood lymphocytes according to standard protocols.

### 2.2 Genetic analyses

Whole exome sequencing of Subject 1 was undertaken as part of a research study at Baylor College of Medicine, whereas Subject 2 had clinical diagnostic exome sequencing at Baylor Genetics Laboratories at Baylor College of Medicine. Exome capture sequencing was conducted using Illumina paired‐end pre‐capture library with the HGSC CORE exome capture design (52 Mb; NimbleGen) and subsequently sequenced on a HiSeq 2500 to an average 20× or greater coverage. Bioinformatic analyses were conducted as previously reported (Stiles et al., 2015). SNVs and small insertions and deletions (InDels) were scored by GATK (DePristo et al., 2011). Quality control filtering of variants was based on coverage, strand bias, mapping quality, and base quality. Custom Perl scripts were used to annotate variants as previously described (Bonnen et al., 2013). Algorithms used for prediction of potential functional consequences of variants included CADD (Kircher et al., 2014), SIFT (Ng & Henikoff, 2002), PolyPhen2 (Adzhubei et al., 2010), Genomic Evolutionary Rate Profiling (GERP) (Davydov et al., 2010), and PhyloP (Cooper et al., 2005).

The candidate *ISCA2* variant identified through exome sequencing was orthogonally validated and recessive segregation through the subjects’ pedigrees was confirmed using PCR‐based Sanger sequencing.

All genetic alleles studied were annotated in reference to *ISCA2* NM_194279.2 for cDNA and NP_919255 for protein. The *ISCA2* NM_194279.2 c.229G>A; p.Gly77Ser variant has ClinVar Variation ID 190396 https://www.ncbi.nlm.nih.gov/clinvar/variation/190396/.

### 2.3 Primary fibroblast and glioblastoma cell culture

Fibroblasts were grown in DMEM High Glucose (Hyclone) supplemented with 15% FBS. Glioblastoma cells T98G were obtained from the American Type Culture Collection (Manassas, VA) and grown in complete Eagle's minimum essential medium (ATCC) supplemented with 10% FBS. For nucleoside rescue experiments, fibroblasts were grown in DMEM supplemented with 1% FBS (low serum medium, LSM), in absence or presence of 150 μM dNTP mix, for 10 days. LSM was replaced with fresh medium with or without dNTPs every 2 days. After 10 days, cells were harvested.

### 2.4 Assessment of mitochondrial membrane potential

Mitochondrial membrane potential was assessed as previously described (Bonnen et al., 2013). Briefly, cells were grown to confluence, counted and stained directly with DilC1(5) or pre‐incubated with carbonyl cyanide 3‐chlorophenylhydrazone (CCCP) prior to staining (MitoProbe DilC1(5) Assay kit for Flow Cytometry; Life Technologies). Results are reported as the difference between the mean fluorescence intensity of cells that received treatment with CCCP versus those that did not. Assays were conducted in triplicate and are shown as the average and standard deviation.

### 2.5 Immunoblotting

Immunoblotting for components of the ETC was conducted on crude mitochondrial extracts prepared from fibroblasts grown under normal conditions. Cell pellets were re‐suspended in ice‐cold isolation buffer (20 mM HEPES pH 7.5, 10 mM KCl, 1.5 mM MgCl2, 1 mM EDTA, 1 mM EGTA, 250 mM sucrose) and allowed to swell for 20 min on ice. The cell suspension was briefly homogenized and then centrifuged for 5 min at 1,000*g* at 4°C. Mitochondria were sedimented from the supernatant by centrifugation at 10,000*g* for 15 min at 4°C. The mitochondria pellet was washed once with ice‐cold isolation buffer and then resuspended in RIPA buffer containing 50 mM Tris, pH 8.0, 150 mM NaCl, 0.5% sodium deoxycholate, 1% Triton X‐100, 0.1% SDS, 5 mg/ml leupeptin, 2 mg/ml aprotinin, and 0.5 mM PMSF. The samples were incubated on ice for 15 min and the lysates were centrifuged at 20,000*g* for 30 min at 4°C. Ten micrograms of mitochondrial lysates was resolved by SDS‐PAGE and transferred to a 0.45 mm PVDF membrane (Millipore). The membrane was immunoblotted using antibodies specific for subunits of the human mitochondrial OXPHOS complexes: NDUFB8, SDHA, UQCRC2, and COX1 (Abcam). The membrane was next incubated in stripping buffer (62.5 mM Tris, pH 6.8, 2% SDS, 100 mM 2‐mercaptoethanol) for 15 min at 50°C, and then probed with TOMM20 (Abcam) as mitochondrial protein loading control. ImageJ software was used to perform the relative quantification of the bands with TOMM20 used for normalization.

Immunoblotting for ABAT (Abcam), ISCA2 (Invitrogen), porin (Abcam), and Lipoic Acid (Calbiochem) was conducted in patient fibroblasts and in T98G glioblastoma cells expressing shRNA targeting ABAT and ISCA2, as well as a non‐target shRNA control. Twenty micrograms of whole cell extract, prepared by standard methods, was resolved on SDS‐PAGE and transferred to a 0.45 mm PVDF membrane (Millipore). ImageJ software was used to perform the relative quantification of the bands with actin used for normalization.

### 2.6 Microscale oxygraphy

Fibroblasts were cultured for 10 days in DMEM containing 25 mM galactose and supplemented with 15% FBS. XF24 extracellular flux analyzer from Seahorse Biosciences was used to measure the rates of oxygen consumption. Cells were plated the previous day of experiment on the XF24 cell culture microplates (Seahorse Biosciences) at a density of 30,000 cells per well. XF24 cartridge (Seahorse Biosciences) was equilibrated with the calibration solution (Seahorse Biosciences) overnight at 37°C. XF assay medium (5 mM galactose, 2 mM Pyruvate) in XF base media (Seahorse Biosciences) was prepared and pH adjusted to 7.0 on the day of the experiment. Oxygen consumption rates (OCRs) were measure for 3 min with 3 min of mixing and 2 min waiting period after each injection of individual cellular stress reagents in the order listed here: 500 nM Oligomycin, 500 nM FCCP, 100 nM Antimycin, and 100 nM Rotenone (Sigma–Aldrich). After the assay was completed, cells in each well were counted using ViCell cell viability analyzer (Beckman Coulter) and the counts were used to normalize the OCRs.

### 2.7 Mitochondrial morphology

Fibroblasts were seeded on micro‐Slide VI flat for Live Cell Analysis (Ibidi). Cells were incubated with 3 μl/ml PicoGreen (Life Technologies) for 60 min then incubated with 50 nM MitoTracker Red CMXRos for 10 min. Next, the staining solution was replaced with fresh growth medium and the cells incubated 20 min. Cells were visualized at 60× magnification using a Nikon Eclipse 90i microscope equipped with TRITC and FITC filters. The images were processed with NIS‐elements v3.0 software.

### 2.8 Quantitation of mtDNA copy number

Mitochondrial genome copy number was determined by real‐time quantitative PCR as previously described with minor modification (Bonnen et al., 2013). Briefly, the mitochondrial genome copy number was determined relative to the nuclear genome using a region of *MTND1* to represent the mitochondrial genome and *B2M* representing the nuclear genome. The assay utilized StepOne Plus RT‐PCR system (Applied Biosystems) and PerfeCTa SYBR Green FastMix ROX (Quanta Biosciences). The assay was performed in triplicate. mtDNA content (mtDNA/B2M ratio) was calculated using the formula: mtDNA content = 1/2^ΔCt^, where ΔCt = Ct^mtDNA^−Ct^B2M^.

### 2.9 shRNA knockdown of ABAT and ISCA2

To knockdown ABAT and ISCA2 expression, T98G glioblastoma cells were transfected with specific targeting shRNA (ABAT: Mission shRNA TRCN0000034927, ISCA2: Mission shRNA Sigma–Aldrich) and a non‐target shRNA control (SHC016; Sigma–Aldrich) using Lipofectamine 2000 (Life Technologies). Forty‐eight hours after transfection, puromycin was added to the culture medium to select transfected cells. After 7 days selection, the cells were allowed to recover and grown in normal media for 3 more days. Cells were harvested and total DNA, RNA, and protein were isolated using standard techniques.

### 2.10 Quantitation of mRNA

RNA extraction from T98G cells expressing non‐targeting control shRNA, ABAT shRNA, and ISCA2 shRNAs was performed using TRIzol reagent (Life Technologies) according to manufacturer's instructions. After extraction, the RNA was treated with RQ1 RNase‐free DNase for 40 min at 37°C. The RNA was then purified using PureLink RNA mini kit (Life Technologies). After resuspension in UltraPure Distilled water (Life Technologies), the RNA was quantified using Quant‐iT RiboGreen RNA assay kit (Life Technologies). One microgram of RNA was used as template for first‐strand cDNA synthesis using SuperScript III First‐Strand Synthesis System for RT‐PCR (Life Technologies) and oligo‐dT according to manufacturer's instructions. Quantitative real‐time PCR experiments were performed using a StepOne Plus RT‐PCR system (Applied Biosystems). RT^2^ qPCR Primer Assays for Human GAPDH and ABAT were purchased from SABiosciences. Oligonucleotides for ISCA2 were purchased from Sigma–Aldrich (F: 5′‐tcacagacagttgcgtcca‐3′ and R: 5′‐gtcgtcggggttgataactg‐3′). PerfeCTa SYBR Green FastMix ROX was purchased from Quanta Biosciences. One microliter of cDNA template was used per reaction (20 μl). Each sample was run in triplicate. The cycling conditions were as follow: 95°C for 3 min (95°C, 15 sec–60°C, 1 min) repeated for 35 cycles. The absence of primer dimers and non‐specific amplification products was confirmed by performing melting curve analysis. The cycle of threshold value (Ct) was normalized to the transcripts for the housekeeping gene GAPDH.

### 2.11 ETC enzymatic activity

Total protein was harvested from cells by standard methods. Briefly, cells were washed in PBS and trypsinized for 3 min at 37°C. Washed two more times in PBS and pellet collected each time. Pellets were sonicated in sonication medium (100 mM Tris, 2 mM EDTA, 250 mM sucrose, pH 7.4) for 5 sec at 60% power on a Misonix Microson XL200 Ultrasonic Cell Disruptor. Protein concentration was determined using Pierce BCA protein assay kit and samples adjusted to 1 mg/μl to perform spectrophotometric kinetic assays. Absorbances were read on a Synergy II microplate reader (Biotek). Enzymatic activity of complexes I–IV and CS were measured as previously published (Kirby, Thorburn, Turnbull, & Taylor, 2007). Complex I activity was determined by measuring rotenone sensitive NADH oxidation (340 nm), complex II activity by measuring DCIP reduction (600 nm), complex III activity by measuring cytochrome *c* reduction (550 nm), complex IV activity by measuring oxidation of reduced cytochrome *c*. Citrate synthase activity by measuring the color of TNB (412 nm), which is generated from DTNB present in the reaction of citrate synthesis, and caused by the deacetylation of Acetyl‐CoA.  Activities were calculated as nmol/min/mg protein. All assays were conducted in triplicate and expressed as percentage of control.

### 2.12 GABA transaminase enzymatic activity

T98G cells expressing non‐targeting control shRNA, ABAT shRNA, and ISCA2 shRNAs were harvested and GABA‐T enzymatic activity was determined using a GABA‐T assay kit (Biomedical Research Service Center, Buffalo University, NY) according to manufacturer instructions. Briefly, the assay is based on sequential GABA‐T transamination reaction and glutamate dehydrogenase reaction, which couples the reduction of iodonitrotetrazolium to iodonitrotetrazolium‐formazan (*Ɛ* = 18 mM^−1^ cm^−1^ at 492 nm). Each sample (10 μg) was assayed in duplicate in a 96‐well plate: one set for control wells (no substrate) and one set for reactions wells (containing GABA‐T substrate). After the incubation periods, the reactions were terminated by adding 3% acetic acid (Sigma–Aldrich, St. Louis, MO). The OD at 492 nm was measured using a plate reader (Phenix Sunrise series; Tecan). Readings were averaged and control wells readings were subtracted from sample wells readings (ΔOD). GABA‐T activity was calculated using the following formula: GABA‐T activity (μmol/(l min) = (ΔOD × 1,000 × 155 μl)/(60 min × 0.6 cm × *Ɛ* × 10 μl) = ΔOD × 23.92, where 155 μl is the total reaction volume, 0.6 cm the light path in the 96‐well plate, 10 μl the volume of sample in each well. The results were graphed as percentage of control.

## 3 RESULTS

### 3.1 Clinical description

**Figure 1:**
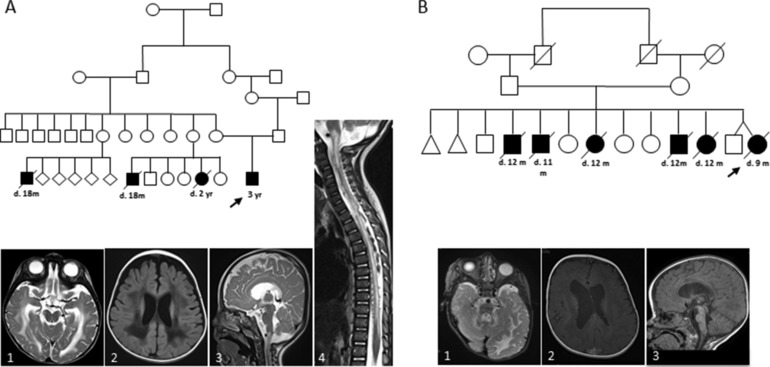
Pedigrees and cranial MRI for ISCA2 deficiency subjects. **A**: Pedigree of Subject 1 revealed maternal family history for fatal infantile leukodystrophy (filled symbols indicate affected individual). MRI of Subject 1 identified high T2 signal abnormalities in white matter indicating leukodystrophy which also spanned the entire length of the spinal cord (1–4). **B**: Subject 2 family history was positive for consanguinity and a history of early infantile leukodystrophy fatality (filled symbols indicate affected individual). Axial T2‐weighted images and Axial T1 images of proband show hyperintensities in the central and periventricular white matter (1–3)

Subject 1 is a male of Saudi Arabian origin with a family history significant for consanguinity and maternal family history positive for leukodystrophy (Figure 1A). The subject's early development was normal until 3 months of age when he had nystagmus and stiff legs. He never sat and at 6 months, there was motor regression with loss of social interaction. Examination at 1 year and 2 months of age revealed signs of central hypotonia with four limb spasticity but normal head circumference. There was optic atrophy with no visual interaction and absent visual evoked potentials. Bulbar dysfunction was significant and nasogastric feeding was required. Brain MRI revealed high T2 signal abnormalities in the white matter including subcortical U fibers and periventricular white matter, extending into the posterior limbs of the internal capsule, posterior body and splenium of the corpus callosum and cerebral peduncles. Abnormal signal was also demonstrated within the dorsal tegmental tracts, inferior cerebellar peduncles and cerebellar white matter. There was restricted diffusion within the dorsal pontine tracts and inferior cerebellar peduncles and abnormal T2 signal change from the craniocervical junction to T11 cord. Brain magnetic resonance spectrometry (MRS) identified an elevated lactate peak. This combination of leukodystrophy of brainstem and spinal cord with elevated lactate is very similar to findings in subjects with recessively inherited *DARS2* mutations though subtle differences in the precise pattern of signal abnormalities in the brainstem and spinal cord may distinguish these two genetic etiologies (Steenweg et al., 2011).

Biochemical testing of cerebrospinal fluid (CSF) revealed elevated glycine (43.0 μmol/l; range: 1.9–10) elevated glutamate (18 μmol/l; range: 0–3.9) and low 5‐methyltetrahydrofolate (46 nmol/l; range: 72–305). Glycine and glutamate levels in plasma were normal; likewise for other plasma amino acid levels. Urine values of glycine and glutamate were elevated (glycine = 2,393 μmol/l; range: 92–760) (glutamate = 54 μmol/l; range: 0–32). While CSF lactate was elevated (lactate = 6.69 μmol/l; range 1.1–2.2), plasma values were normal (lactate 1.84 μmol/l; range 0.7–2.1). Additional metabolic findings included elevated plasma chitotriosidase activity (500 nmol/hr/ml; range: 0–150), decreased palmitoyl protein thioesterase activity (12 nmol/hr/mg ptn; range: 17–139), decreased β‐galactosidase activity (143 nmol/hr/mg ptn; range 163–378), and decreased arylsulfatase A activity (17 nmol/hr/mg ptn; range: 22–103). At time of reporting, the subject was still alive, and was most recently reviewed clinically at age 3 years 5 months (Table 1).

**Table 1:** Summary of patient clinical features

	Subject 1	Subject 2
*ISCA2* variant (NM_194279.2)	c.229G>A p.Gly77Ser	c.229G>A p.Gly77Ser
Gender	Male	Female
Age of onset	3 m	6 m
Current age	3 y 5 m	Deceased
Age at last clinic visit	1 y 9 m	9 m
Age at death	N/A	9 m
Growth		
Poor growth	+	+
Poor weight gain	+	−
Head circumference	50th centile	Relative macrocephaly
Ophthalmologic		
Optic nerve pallor	+	+
Visual impairment	+	+
Dysmorphic features		
Low set ears	+	−
Broad nasal bridge	+	−
Respiratory		
Frequent infections	−	−
Tracheostomy	−	−
Gastrointestinal		
Feeding difficulties/aspiration	+/−	+/+
Constipation	−	+
Nasogastric tube	+	−
Skeletal		
Joint laxity	−	+
Short fourth metacarpals	−	+
Cutaneous toe syndactyly	−	+
Neurological		
Development delays	+	+
Developmental regression	+, 6 m	+, 6 m
Expressive language	−	−
Hypotonia	+, truncal	+, truncal
Spasticity	+	+
Abnormal movements	−	−
Ambulation	−	−
Seizure	−	−
Radiological		
Leukodystrophy	+	+
MRS	Elevated lactate peak	Elevated lactate peak
T1/T2 signal abnormalities	Increased T2 in WM of SUF, PVWM extending into PLIC, PB, and SP of CC and CP.	Diffuse T1 and T2 in WM of FP, OC, and both CBH
	Abnormal signal DTT, ICP, and CWM	
	Abnormal T2 in CCJ to T11 cord	
Metabolic		N/A
CSF		
Glycine	43.0 μmol/l (1.9–10)	
Glutamate	18 μmol/l (0–3.9)	
5‐MET	46 nmol/l (72–305)	
Lactate	6.69 mmol/l (1.1–2.2)	
Plasma		
Glycine	Normal	
Glutamate	Normal	
Other PAA	Normal	
Lactate	1.84 mmol/l (0.7–2.1)	
Chitotriosidase	500 nmol/hr/ml (0–150)	
Palmitoyl thioesterase	12 nmol/hr/mg (17–139)	
B‐glucosidase	143 nmol/hr/mg (163–378)	
Arylsulfatase A	17 nmol/hr/mg (22–103)	
Urine		
Glycine	2,393 μmol/l (92–760)	
Glutamate	54 μmol/l (0–32)	

m, months; y, years; N/A, not applicable; MRS; functional magnetic resonance spectrometry; WM, white matter; SUF, subcortical U fibers; PVWM, periventricular white matter; PLIC, posterior limb of the internal capsule; PB, posterior body; SP, splenium; CC, corpus callosum; CP, cerebral peduncles; DTT, dorsal tegmental tracts; ICP, inferior cerebellar peduncles; CWM, cerebellar white matter; CCJ, craniocervical junction; FP, frontoparietal; OC, occipital; CBH, cerebellar hemispheres; CSF, cerebrospinal fluid; 5‐MET, 5‐methyltetrahydrofolate; PAA, plasma amino acids.

Subject 2 is a female of Saudi Arabian origin who presented to clinic at age 6 months with diffuse hypotonia and developmental regression. Family history was significant for parental first‐cousin consanguinity, two miscarriages and five siblings who died in infancy from demyelination and white matter disease associated with regression (Figure 1B). Her development was normal until about 6 months of age when she stopped making sounds, diminished eye contact, lost the ability to roll and became unable to move. Further evaluations at 9 months of age revealed spasticity, optic nerve pallor and visual impairment. Brain MRI at this time showed leukodystrophy with diffuse T1 and T2 signal abnormalities in the white matter, in the frontoparietal, occipital, and both cerebellar hemispheres (Figure 1B). Brain MRS identified a large lactate peak. Additional features included poor growth with relative macrocephaly, and skeletal abnormalities including joint laxity, short fourth metacarpals and cutaneous toe syndactyly. Skeletal abnormalities have not been reported in the previous *ISCA2* deficiency subjects. Subject 2 died aged 9 months. No metabolic testing was conducted.

### 3.2 Genetic analyses

Exome sequencing was conducted on Subject 1 and Subject 2 and both individuals were found to be homozygous for *ISCA2* variant, c.229G>A; p.Gly77Ser, that was recently reported pathogenic in a group of six individuals from five independent consanguineous families with similar clinical presentation (Al‐Hassnan et al., 2015). Sequencing of the parents of each proband showed all were heterozygous for the *ISCA2* variant and confirmed recessive inheritance in the families.

### 3.3 ISCA2 mutation results in deficiency of mitochondrial bioenergetics

**Figure 2:**
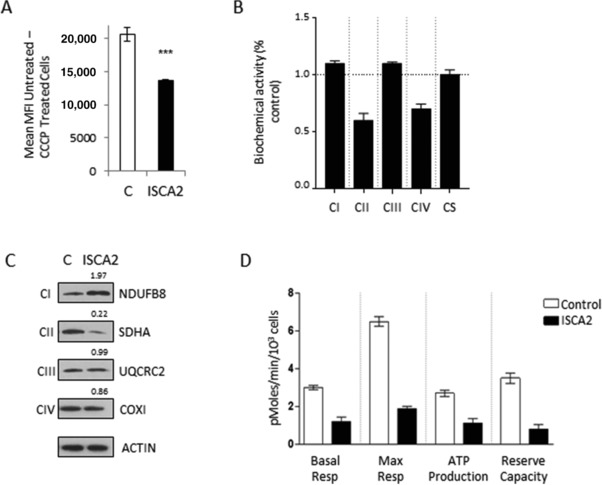
*ISCA2* variant alters mitochondrial bioenergetics in subject fibroblasts. **A**: Mitochondrial membrane potential is significantly diminished in ISCA2 deficiency subject fibroblast cell line. **B**: Assessment of electron transport chain complex activity of ISCA2 deficiency subject fibroblast revealed mildly elevated activity for complexes I and III, whereas complex II and IV were significantly reduced. **C**: Protein quantification by immunoblotting shows complex I (NDUFB8) was elevated in patient cells compared with control cells, whereas complex II (SDHA) is diminished. TOMM20 was used a mitochondrial marker and loading control. **D**: Microscale oxygraphy analysis of live fibroblasts demonstrated a profound respiratory deficiency in ISCA2 subject fibroblasts (black bars), compared with healthy control fibroblast lines (white bars). Error bars for all data indicate standard deviation. ***P < 0.001

Mitochondrial membrane potential is the electrical gradient across the inner mitochondrial membrane generated by the reductive transfer of electrons through the ETC protein complexes. This reflects cellular capacity to generate ATP by oxidative phosphorylation and as such, is an indicator of mitochondrial and cellular health. The membrane potential of dermal fibroblasts from Subject 1 was significantly diminished relative to control (*P *< 0.001) indicating mitochondrial dysfunction (Figure 2A).

We next directly assessed the biochemical activity of each of ETC complexes I, II, III, and IV plus citrate synthase in fibroblasts from Subject 1. The activity of complexes I and III were marginally increased, whereas complexes II and IV were markedly decreased to 60% and 70% of control respectively (Figure 2B). Citrate synthase was the same as control fibroblasts. While the decreases in activity of complexes II and IV were significant, neither was sufficient to meet the Modified Walker diagnostic criteria. An additional assessment of capacity for oxidative phosphorylation in fibroblasts from Subject 1 was conducted by immunoblotting for specific components of each complex of the ETC. Similar to biochemical activity, this revealed an increase in Complex I subunit NDUFB8 and a significant decrease in Complex II subunit SDHA. In addition, a marginal decrease was observed for Complex IV subunit protein COX1 (Figure 2C).

OCRs were measured in Subject 1 fibroblasts using microscale oxygraphy. This measurement of respiration in live cells revealed significantly diminished oxygen consumption and ATP production in Subject 1 (Figure 2D). Basal respiration was severely compromised relative to control as was maximal respiration suggesting a defect in electron flow from complexes I–IV. ATP production was reduced to nearly half of control. In addition, spare respiratory capacity was only 25% of control suggesting cells are likely unable to accommodate additional energetic demand (Figure 2D). Taken together, our results indicate that the *ISCA2* c.229G > A; p.Gly77Ser variant in the homozygous state severely altered the bioenergetic properties of mitochondria.

### 3.4 *ISCA2* mutation alters mitochondrial morphology and mtDNA copy number

**Figure 3:**
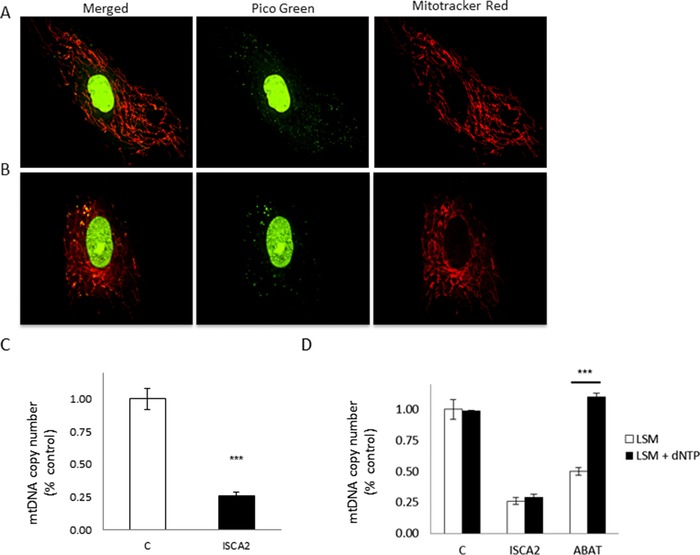
ISCA2 subject fibroblast display mitochondrial network disruption, severe mtDNA depletion and lipoylation defects. **A** and **B**: Images of control fibroblasts (**A**) display a ubiquitous mitochondrial distribution and networking by MitoTracker Red staining and a typical distribution of mtDNA nucleoids by PicoGreen staining, whereas Subject 1 cells (**B**) display blunted mitochondrial networking with an abnormal perinuclear distribution. **C**: Profound mtDNA depletion was determined in *ISCA2* subject fibroblasts relative to controls. **D**: Quantification of mtDNA copy number in quiescent subject fibroblast lines. ABAT subject fibroblasts show a reduced mtDNA copy number in low serum media (LSM), but mtDNA copy number is rescued with the addition of dNTPs. ISCA2 deficiency subject fibroblasts show reduced mtDNA copy number in LSM media, and this remains unchanged despite the addition of dNTPs. Error bars indicate standard deviation. ***P < 0.001

To further assess the role of altered ISCA2 function in mitochondrial physiology, we examined the morphology and distribution of the organelle. Mitotracker Red staining of mitochondria in Subject 1 cells revealed an abnormal distribution and shortening of the mitochondrial network similar to what is observed in some mtDNA maintenance disorders (Figure 3A and B). Quantitation of mtDNA copy number revealed cells retained only 25% of mtDNA content relative to control (Figure 3C).

### 3.5 mtDNA depletion observed in ISCA2 subject tissues is not caused by defects in the mitochondrial nucleoside salvage pathway

*ISCA2* deficient subject fibroblasts displayed mtDNA depletion and the mechanism for this is unknown (Figure 3C). Multiple pathways contribute to maintenance of mtDNA including replication and nucleoside maintenance. *ABAT* (MIM#137150) encodes a dual‐function 2Fe–2S mitochondrial enzyme that is responsible for the catabolism of GABA and plays a role in the maintenance of mtDNA via its role in the mitochondrial nucleoside salvage pathway (Besse et al., 2016; Besse et al., 2015; Storici et al., 2004). If ISCA2 played a role in 2Fe‐2S iron cluster maturation or transfer, the mtDNA depletion could be in part due to a reduction in function of the 2Fe–2S mitochondrial protein, ABAT. To test whether *ABAT* function in this pathway is altered, we performed nucleoside rescue assays. Subject primary cell lines were cultured in LSM to induce G0 where DNA replication is halted, but mitochondria continue to replicate and the mitochondrial nucleoside salvage pathway is most heavily relied upon for replication of mtDNA. Cells were either supplemented with dNTPs or not and measured for mtDNA copy number. Healthy control fibroblasts displayed no change in mtDNA copy number under either condition (Figure 3D). As expected, *ABAT* deficiency subject fibroblast cultured in LSM displayed a significant reduction in mtDNA copy number, but the mtDNA copy number was rescued with addition of dNTPs to the media (Figure 3D). *ISCA2* Subject 1 fibroblasts displayed significantly reduced mtDNA copy number in LSM, however this effect was unchanged with the addition of dNTPs (Figure 3D). Our data demonstrate that the reduction of mtDNA copy number in these patients is not due to a defect in the 2Fe–2S protein ABAT or any defect in the mitochondrial nucleoside salvage pathway.

### 3.6 ISCA2 depletion results in defects in 4Fe–4S‐dependent enzymes and not 2Fe–2S‐dependent enzymes

**Figure 4:**
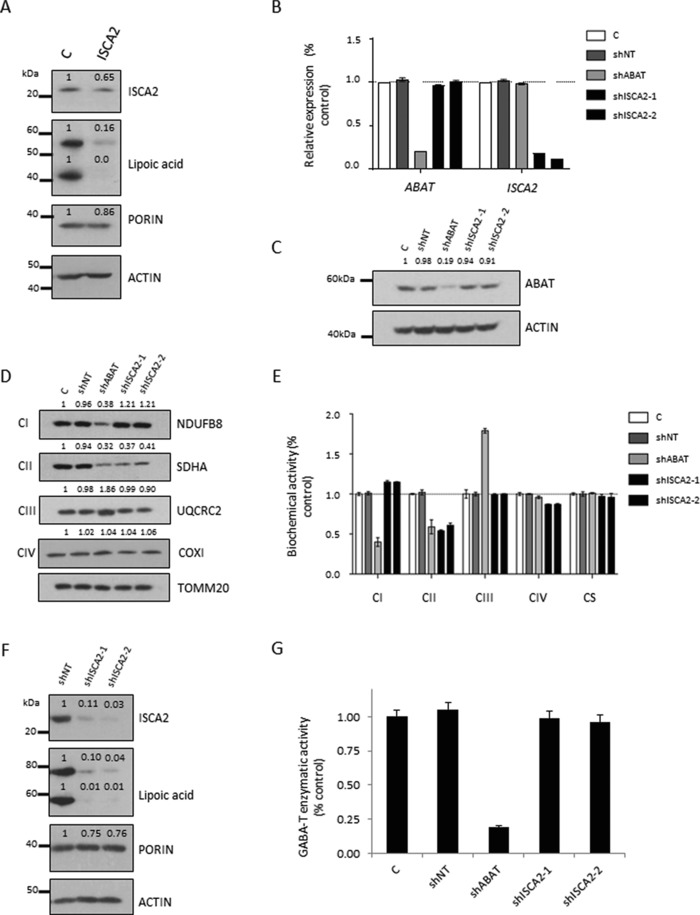
Characterization of *ISCA2* depletion model for mitochondrial bioenergetics and lipoylation defects. **A**: Immunoblotting of Subject 1 fibroblasts revealed reduced ISCA2 levels. Immunoblotting for lipoic acid bound proteins found severely reduced lipoylated protein in Subject 1 fibroblasts relative to controls. **B**: qRT‐PCR analysis of T98G glioblastoma cells expressing control non‐targeting shRNA (NT shRNA), ABAT shRNA (shABAT), and two independent ISCA2 shRNAs (shISCA2‐1 and shISCA2‐2). **C**: Immunoblotting of ABAT across each depletion line displayed a significant reduction in protein for cells expressing ABAT shRNA, but not for cells expressing either ISCA2 shRNA. **D**: Significantly reduced protein levels were observed in ABAT depleted cells for complex I (NDUFB8) and II (SDHA) proteins, but complex III (UQCRC2) protein levels were significantly elevated. Both ISCA2 depletion cells showed unchanged complex I (NDUFB8), III (UQCRC2), and IV (COXI) protein levels, but significantly reduced complex II protein levels. TOMM20 was used as a mitochondrial marker and loading control. **E**: Respiratory chain complex biochemical activity was determined in depletion model cells. ABAT depleted cells showed significantly reduced complex I and II levels, but elevated complex III activity. Cells transduced with either ISCA2 shRNA displayed significantly reduced complex II activity, but relatively unchanged activity for complexes I, III, and IV. Citrate synthase (CS) was unchanged across all samples. **F**: Cells expressing either ISCA2 shRNA displayed significantly reduced ISCA2 protein levels and lipoic acid bound proteins. **G**: GABA transaminase activity was significantly reduced in cells expressing ABAT shRNA, but not in cells expressing either ISCA2 shRNA. Error bars indicate standard deviation. ***P < 0.001

Hyperglycinemia is one of the clinical features in our patient that is less commonly observed with mitochondrial encephalopathies. Hyperglycinemia often results from dysfunction of the glycine cleavage system, the proper functioning of which requires the 4Fe–S cluster enzyme lipoic acid synthase (LIAS)(Harmer et al., 2014). Previous studies have shown that individuals with deficiency of LIAS (Mayr et al., 2011) or components of the Fe–S cluster machinery NFU1 (Ahting et al., 2015; Cameron et al., 2011; Navarro‐Sastre et al., 2011), BOLA3 (Ahting et al., 2015; Haack et al., 2013), IBA57 (Debray et al., 2015), and GLRX5 (Baker et al., 2014; Wei, Weng, Lee, Hwu, & Lee, 2011) displayed hyperglycinemia as was observed in our ISCA2 patient. We investigated whether ISCA2 subjects may have reduced lipoylation resulting from the primary defect in Fe–S cluster generation. Immunoblotting for lipoic acid in Subject 1 fibroblasts showed dramatically reduced levels of protein lipoylation in Subject 1 relative to controls (Figure 4A) indicating a defect in the production of lipoic acid presumably due to faulty Fe–S cluster generation derived from mutant ISCA2.

To further examine the role of ISCA2 in the Fe–S cluster machinery and specifically in the role of mitochondrial 2Fe–2S versus 4Fe–4S‐dependent enzymes, we generated two independent *ISCA2* knockdown cell lines by stable transduction of two different *ISCA2* shRNA. Quantitation of ISCA2 mRNA in each *ISCA2* knockdown cell line showed ISCA2 mRNA and protein levels reduced to below 15% of control (Figure 4B and F). We utilized these cell lines to study the activity of multiple enzymes that are 2Fe–2S (UQCRC2, ABAT) or 4Fe–4S dependent (LIAS). Protein components of ETC complexes I and II contain both 2Fe–2S and 4Fe–4S clusters, whereas complex III relies only on a single 2Fe–2S cluster.

Quantitation of respiratory subunits by immunoblotting in *ISCA2* knockdown cell lines revealed levels of complex I subunit protein NDUFB8 were marginally increased and complex III subunit UQCRC2 levels were unchanged. Notably, a significant decrease of complex II subunit SDHA was observed across both *ISCA2* shRNA lines (Figure 4D). These results mirror those observed in Subject 1 fibroblasts. Enzymatic activity for complexes I–IV in both *ISCA2* depletion lines displayed activities consistent with immunoblotting results; the primary difference from control was significantly reduced complex II activity, with mild changes in complex I and IV activity (Figure 4E). In addition, protein lipoylation was measured in each *ISCA2* knockdown line. Immunoblotting of lipoic acid bound protein in each depletion line mimicked our observations in subject fibroblasts with severely diminished level of protein lipoylation relative to controls (Figure 4F).

Since deficiencies in one ETC complex can influence the performance of other ETC complexes even when a primary defect is not present, as a control we also generated an *ABAT* shRNA cell line that had ABAT mRNA and protein levels measuring ∼20% of control (Figure 4B and C). In contrast to *ISCA2* knockdown cell lines, ABAT depletion cell lines displayed significantly reduced complex I and II protein levels and activity alongside elevated complex III protein and activity (Figure 4D and E). In addition, ABAT protein and enzymatic activity levels were directly tested to represent the 2Fe–2S class of mitochondrial proteins in addition to ubiquinol‐cytochrome *c* reductase complex (UQCRC or complex III). Both the ABAT protein levels (Figure 4C) and GABA transaminase enzymatic activity levels (Figure 4G) were normal in the ISCA2 shRNA lines consistent with the idea that ISCA2 is dispensable for ABAT function. Taken together these experiments demonstrate significant deficits in the functioning of 4Fe–4S‐dependent proteins, LIAS, without deficiency of 2Fe–2S proteins as exemplified by ABAT.

## 4 DISCUSSION

We present two new cases of *ISCA2* deficiency caused by homozygous presence of the *ISCA2* c.229G > A; p.Gly77Ser variant whose clinical presentation is consistent with the infantile‐onset leukodystrophy cases previously described. We provide additional phenotypic information through a more comprehensive clinical description and studies on patient cells and *ISCA2* knock‐down cell models. We identified a profile of metabolic dysfunction that includes elevated CSF glycine and glutamate alongside low 5‐methyltetrahydrofolate. Radiological findings showed a combination of features, most notably, leukodystrophy of the brainstem with longitudinally extensive spinal cord involvement and elevated lactate, and illustrate the pleiotropic effects of abnormal *ISCA2* gene function. In addition, these radiological features are similar to findings in subjects with *DARS2* mutations, though not identical (Steenweg et al., 2011). Functional studies demonstrated that loss‐of‐function of ISCA2 impairs respiration, ATP production, mitochondrial membrane potential, and results in shortened mitochondrial network and diminished mtDNA copy number. Moreover, we demonstrate that disruption of *ISCA2* does not modify the function of the 2Fe–2S‐dependent mitochondrial proteins ABAT and UQCRC2, but does diminish the function of ETC complex II and protein lipoylation, which is dependent on 4Fe–4S proteins. These discoveries support the notion that ISCA2 likely functions in 4Fe–4S cluster assembly, which consequently affects a diverse array of cellular processes including proper functioning of the mitochondria.

Metabolic findings revealed a signature of elevated glycine, glutamate, lactate, and pyruvate in CSF, whereas all showed normal values in plasma. Plasma or CSF glycine was not assessed in previously reported ISCA2 patients (Al‐Hassnan et al., 2015; Lebigot et al., 2017). Subjects with bi‐allelic pathogenic variants in another gene essential for Fe–S cluster assembly, *IBA57*, also show elevated glycine in CSF and plasma (Ajit Bolar et al., 2013; Debray et al., 2015). These observations of elevated glycine puts Fe–S cluster deficiency syndromes in the differential for a group of disorders that result in hyperglycinemia. Elevated glycine is observed in the CSF of subjects with glycine encephalopathy (CSF glycine > 80 for neonatal form; glycine > 30 for milder “atypical form”) due to recessive mutations in the glycine cleavage system pathway genes *GLDC* (MIM# 238300), *AMT* (MIM# 238310), and *GCSH* (MIM# 238330) (Applegarth & Toone, 2001; Nanao et al., 1994; Toone, Applegarth, Coulter‐Mackie, & James, 2000). While these gene products are not reliant on Fe–S clusters for proper functioning, they do require lipoic acid, which is generated by 4Fe–4S‐dependent enzymes (Hiltunen et al., 2010). In our study, we observed a sizeable reduction in lipoylated proteins in both subject fibroblasts and *ISCA2* depletion models, suggesting that the 4Fe–4S‐dependent enzyme LIAS is adversely affected, and consequently lipoic acid dependent enzymes such as those involved in the glycine cleavage system and mitochondrial energy metabolism. Individuals with pathogenic variants in other Fe–S cluster proteins, NFU1, BOLA3, IBA57, and GLRX5 have elevated glycine levels suggesting that the phenotypic overlap of Fe–S deficiencies with LIAS may be due to alterations in glycine cleavage (Tort, Ferrer‐Cortes, & Ribes, 2016). Another group of metabolic disorders, such as propionic acidemias, can cause ketotic hyperglycinemia, which produces elevated glycine in plasma and urine, however, not typically in CSF (Gerritsen, Kaveggia, & Waisman, 1965).

Protein levels and enzymatic activity assays conducted in both patient tissue and knockdown cell models demonstrated decreased Complex II with intact complexes I and III. Complexes I, II and III are structurally composed of multiple subunits that contain a range of Fe‐S clusters. Complex I contains a total of eight Fe/S clusters (two 2Fe–2S and six 4Fe–4S), which are found in subunits NDUFS1, NDUFS7, NDUFS8, NDUFV1, and NDUFV2. Complex II utilizes three Fe‐S clusters, one each of 2Fe–2S, 3Fe–4S, and 4Fe‐4S, whereas complex III carries a single 2Fe–2S cluster (Lill and Mulenhoff, 2008). In a most simplistic model of ISCA2 playing a role in the maturation of 4Fe–4S clusters but not 2Fe–2S clusters, it would be expected that complexes I and II would show deficiencies and that complex III would not. A previous study using an ISCA2 knockdown model in HeLa cells, observed diminished complex I activity assessed through a Blue Native in‐gel activity assay (Sheftel et al., 2012). This study also found decreased complexes II, III, and IV enzymatic activity and protein levels. Previous studies in patient fibroblasts reported decreased complex I activity based on antibody binding (Al‐Hassnan et al., 2015) and enzymatic activity (Lebigot et al., 2017). The differences between studies may reflect differences between cell types and/or variation due to the various methods utilized to assess function of complexes I–IV.

Our observations of reduced complex II (SDHA) levels and activity (SDH) indicate a role for ISCA2 in the proper functioning of SDH, in concordance with observations that defects in 4Fe–4S cluster assembly impair specific recipient enzymes (Gelling et al., 2008; Maio & Rouault, 2015; Melber et al., 2016; Muhlenhoff et al., 2011; Sheftel et al., 2012). Interestingly, recent studies have shown there may be different mechanisms for delivery and incorporation of 4Fe–4S clusters into SDHB, the sole Fe–S containing subunit of SDH. The study of loss‐of‐function of late‐acting ISCA complex member proteins Nfu1p and Isa2p in yeast showed specific loss of SDH activity and SDHB protein levels (Melber et al., 2016). However, the loss of function of the SDH assembly factor SDHAF1 in mammalian cells also showed diminished SDH activity that was dependent on early‐acting ISCU complex (Maio & Rouault, 2015). The differences between studies may reflect differences between species and/or respiratory states, and the possibility that both complexes play a role in this sophisticated cellular process.

ISCA1/2 yeast orthologs ISA1/2 has been shown to physically interact with IBA57 in *S. cerevisiae* and these three proteins are thought to form a multi‐protein complex that facilitates maturation of 4Fe–4S clusters (Gelling et al., 2008). Recent work has identified two families with *ISCA1* mutations but functional work has yet to be completed (Shukla et al., 2017). Bi‐allelic variants in *IBA57* have been reported in three unrelated families to cause various clinical presentations. One consanguineous family segregating a recessive loss‐of‐function variant in two siblings displayed a severe neonatal‐onset encephalomyopathy characterized by severe hypotonia, generalized muscle weakness, absent primitive reflexes, microcephaly, arthrogryposis, lactic acidosis, hyperglycinemia and perinatal death (OMIM ID 615316)(Ajit Bolar et al., 2013). Fibroblasts from these two siblings showed activity of ETC complex II was diminished and complex III normal, whereas complexes I and IV were not measured. Similarly, in muscle of these subjects, protein levels and activity of complexes I, II, and IV were diminished and complex III was normal. A second consanguineous family with an *IBA57* splice site variant was reported with a notably different clinical presentation of childhood‐onset slowly progressive spastic paraparesis with optic atrophy and peripheral neuropathy referred to as Spastic Paraplegia 74 (OMIM ID 616451) (Lossos et al., 2015). A third consanguineous family segregating a recessive loss‐of‐function variant displayed an intermediate phenotype relative to the other families. The patient was asymptomatic at birth, but by 6 months displayed signs of motor regression including progressive hypotonia and muscle weakness, developmental regression and leukodystrophy resulting in death at 17 months of age (Debray et al., 2015). Fibroblasts from this subject showed reduced complexes I and II with normal complex III activity. In the same study, a IBA57 knockdown cell model showed reduced complex II activity with normal complexes I and III (Debray et al., 2015). A recent study identified reduced IBA57 protein levels in ISCA2 deficiency patient fibroblasts suggesting an interaction between these proteins and their overlapping phenotypes (Lebigot et al., 2017). The compromise in ETC complex activity caused by IBA57 deficiency parallels that of ISCA2 deficiency subjects with the greatest compromise in oxidative phosphorylation observed in complex II, although the precise role of ISCA2 in providing mature 4Fe–4S clusters to the ETC complex proteins remains unresolved.

We showed ISCA2 deficiency subject's fibroblasts have an abnormally short distribution of the mitochondrial network similar to what has been observed in IBA57 deficiency fibroblasts (Ajit Bolar et al., 2013). Analysis of mtDNA copy number identified severely depleted levels, where Subject 1 fibroblast cells retained only 25% of mtDNA content relative to control. This quantitative loss of mtDNA copy number was consistent with previous reports of ISCA2 deficiency subjects (Al‐Hassnan et al., 2015). Primary mitochondrial disorders that include depletion of mtDNA typically also show combined OXPHOS deficiencies involving complexes I and IV with the nuclear‐encoded complex II functionally preserved. The cellular phenotype of having mtDNA depletion together with complex II deficiency along with hyperglycinemia, elevated lactate, leukodystrophy of the brainstem and complete length of the spinal cord is a relatively unusual combination of features that may facilitate diagnosis of ISCA2 deficiency patients in combination with genetic analyses.
